# Angle-resolved stochastic photon emission in the quantum radiation-dominated regime

**DOI:** 10.1038/s41598-017-11871-0

**Published:** 2017-09-14

**Authors:** Jian-Xing Li, Yue-Yue Chen, Karen Z. Hatsagortsyan, Christoph H. Keitel

**Affiliations:** Max-Planck-Institut für Kernphysik, Saupfercheckweg 1, 69117 Heidelberg, Germany

## Abstract

Signatures of stochastic effects in the radiation of a relativistic electron beam interacting with a counterpropagating superstrong short focused laser pulse are investigated in a quantum regime when the electron’s radiation dominates its dynamics. We consider the electron-laser interaction at near-reflection conditions when pronounced high-energy gamma-ray bursts arise in the backward-emission direction with respect to the initial motion of the electrons. The quantum stochastic nature of the gamma-photon emission is exhibited in the angular distributions of the radiation and explained in an intuitive picture. Although, the visibility of the stochasticity signatures depends on the laser and electron beam parameters, the signatures are of a qualitative nature and robust. The stochasticity, a fundamental quantum property of photon emission, should thus be measurable rather straightforwardly with laser technology available in near future.

## Introduction

The next generation petawatt laser systems^1, 2^ will open a door not only to novel regimes of laser-matter interaction^3, 4^, but also to new perspectives for the investigation of fundamental problems^5–9^. In ultrastrong laser fields the quantum properties of electron radiation, the discrete and probabilistic character of photon emission, can be conspicuous. While the discreteness of the radiation photon energy is known to be observed straightforwardly, e.g., in the Compton scattering as a shift of the emission frequency^10–12^, the signatures of the stochastic character of photon emission are more subtle and elaborate for observation. The latter has impact on the radiation back-action to the electron dynamics and should be more apparent in the so-called radiation dominated regime (RDR) of interaction^8, 13, 14^, when multiple emission of photons by an electron becomes probable. One of the conceptual consequences of stochasticity effects (SE) in photon emissions, i.e., the probabilistic nature of photon emission, is the broadening of the energy spread of an electron beam in a plane laser field^15, 16^, while similar effect can cause electron stochastic heating in a standing laser field^17^. However, in an experiment in a focused laser beam, competing effects may arise, e.g., an additional energy spreading of the electron beam due to the difference of radiative losses of electrons in the electron-beam cross section. Another SE signature is the so-called electron straggling effect during radiation in strong fields^18–21^, when the electrons propagate a long distance without radiation due to SE, resulting in the increase of the yield of high-energy photons. However, we will show below that the straggling effect is rather weak in the considered regime. Different signatures of quantum radiation reaction have been discussed^22–35^ which include all quantum effects, such as photon recoil, stochasticity, and interferences^35^. However, they do not isolate information on the specific role of stochasticity. Unfortunately, so far unequivocal SE have not been observed in an experiment, which would also be important as a clear test for the underlying theories. We underline that in this paper SE refers to the quantum probabilistic nature of photon emission, but not to the chaotic classical dynamics as, e.g., in ref. 36.

The quantum effects in strong laser fields are determined by the invariant parameter $\chi \equiv |e|\sqrt{{({F}_{\mu \nu }{p}^{\nu })}^{2}}/{m}^{3}$ ^37, 38^, where *F*_*μν*_ is the field tensor, *p* ^*ν*^ = (*ε*, **p**) the incoming electron 4-momentum, and *e* and *m* are the electron charge and mass, respectively (Planck units *ħ* = *c* = 1 are used throughout). The RDR, when the radiation losses during a laser period are comparable with the electron initial energy, is characterized by the parameter $R\equiv \alpha \xi \chi \mathop{ > }\limits_{ \tilde {}}1$ ^8^, where *α* is the fine structure constant, *ξ* ≡ |*e*|*E*_0_/(*mω*_0_) the invariant laser field parameter, while *E*_0_ and *ω*_0_ are the laser field amplitude and frequency, respectively. In the quantum RDR the stochastic nature of photon emission is a fundamental quantum property and has to be taken into account during the high-energy photon radiation^39–42^, that significantly affects the electron dynamics and the high-energy photon emission.

In this paper, we investigate signatures of the stochastic nature of photon emission in the nonlinear Compton scattering in the quantum RDR during the interaction of a superstrong short focused laser pulse with a counterpropagating relativistic electron beam. We consider the interaction in the electron near-reflection regime^14^, where the front electrons are reflected and emit gamma-rays in the near-backward direction^29^: due to the combined effects of the laser focusing and radiation reaction, the front electrons of the electron beam are reflected and emit ultrashort gamma-rays in the near-backward direction with respect to the initial electron motion^29^. In the considered case with $\chi \mathop{ < }\limits_{ \tilde {}}1$, the straggling effect is rather weak, however, the electron near-reflection regime offers a possibility to observe the SE in the angular distribution of the radiation. In fact, the photon emissions in different laser cycles are essentially modified due to the SE in the RDR, and the latter is mapped into the broad backward-emission angles when the electron is at the near-reflection condition. We calculate angle-resolved radiation intensity and photon numbers and show that, due to the stochastic nature of photon emission, the radiation’s angular distribution (RAD) has a single prominent peak in the backward direction, which is broad and easily observable in an experiment. In contrast, when the SE are ignored, the backward radiation yields an angular distribution with several peaks. Furthermore, we investigate the influences of the laser and electron-beam parameters (the laser focal radius, the laser pulse duration and the electron initial energy) on the visibility of those SE signatures, to optimize parameters for a future experimental setup.

## The setup and radiation spectra

The considered quantum RDR requires the invariant parameters $\chi \equiv \gamma ({\omega }_{0}/m)\xi \mathrm{(1}-\beta \,\cos \,\theta )\approx {10}^{-6}\gamma \xi \mathop{ < }\limits_{ \tilde {}}1$ and $R\equiv \alpha \xi \chi \mathop{ > }\limits_{ \tilde {}}1$, while the electron near-reflection regime does $\gamma \sim \xi /2$, where *γ* is the Lorenz factor of the electron, and *β* the electron velocity scaled by the light speed in vacuum. The two conditions above demand $\gamma \sim \xi \sim {10}^{3}$, i.e., an electron beam of GeV energies and laser intensities of 10^23^–10^24^ W/cm^2^ anticipated in next generation facilities^1, 2^.

The calculation of the radiation is based on Monte-Carlo simulations employing QED theory for the electron radiation and classical equations of motion for the propagation of electrons between photon emissions^40–42^. In superstrong laser fields $\xi \gg 1$, the coherence length of the photon emission is much smaller than the laser wavelength and the typical size of the electron trajectory^38, 43^. Then, the photon emission probability is determined by the local electron trajectory, consequently, by the local value of the parameter *χ* ^44^. The radiation in the quantum regime ignoring the SE are calculated by employing the Sokolov equation^22–24^, when the radiation is emitted continuously along the electron trajectory and modifies accordingly the classical equations of motion. In this work we consider the regime when the quantum invariant field parameter *χ* is not small, $\chi \mathop{ < }\limits_{ \tilde {}}1$. Therefore, the quantum recoil in photon emission is significant even in the case when the probabilistic character (stochasticity) of photon emission is neglected. Therefore radiation intensity and the radiation reaction should be calculated using quantum corrections. The Sokolov equation is a phenomenologically derived equation of motion for an electron in the $\xi \gg 1$ limit, which is based on the energy-momentum conservation within the system of the electron and emitted photons at each formation length of radiation [however, it is argued in ref. 45 that this equation cannot be derived from QED by taking the classical limit]. The Sokolov equation adapts the electron energy-momentum at each step according to the radiated energy-momentum including quantum corrections. It provides a natural way to treat the electron dynamics in the external field classically but to take into account in the radiation-reaction quantum-recoil corrections (but not stochasticity). In contrast, the conceptually more systematic Lorentz-Abraham-Dirac or Landau-Lifshitz equations account for the radiation reaction only classically (i.e. without recoil). The simulation methods including and excluding the SE are described in detail in the section “Methods”.

We employ a linearly polarized tightly focused laser pulse with a Gaussian temporal profile, which propagates along +*z*-direction and polarizes in *x*-direction. The spatial distribution of the electromagnetic fields takes into account up to the *ε* ^3^-order of the nonparaxial solution^46, 47^, where *ε* = *w*_0_/*z*_*r*_, *w*_0_ is the laser focal radius, ${z}_{r}={k}_{0}{w}_{0}^{2}/2$ the Rayleigh length with the laser wave vector *k*_0_ = 2*π*/*λ*_0_, and *λ*_0_ the laser wavelength. The expressions of the laser fields are represented in the section “Methods”.

**Figure 1:**
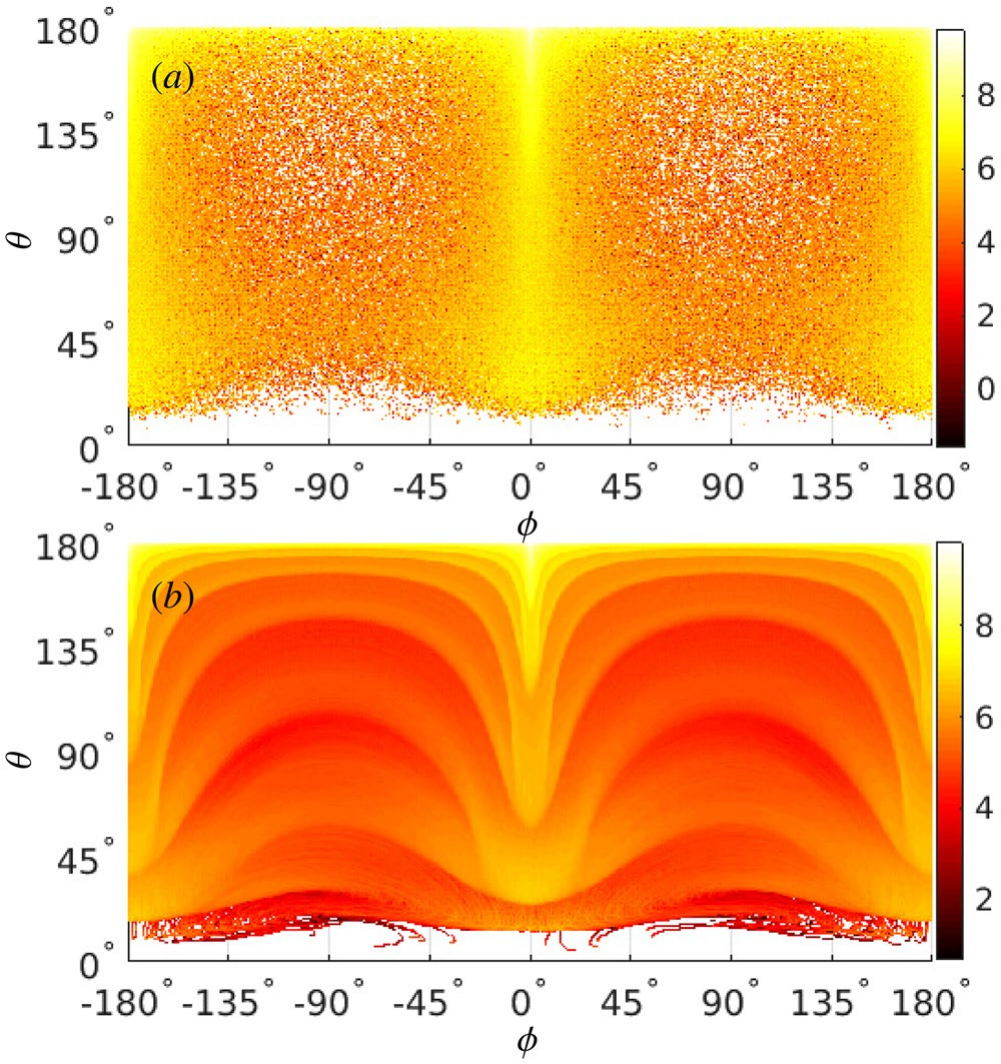
Angle-resolved radiation energy *ε*_*R*_ in units of the electron rest energy *m* vs the emission polar angle *θ* and the azimuthal angle *ϕ*: (**a**) including and (**b**) excluding SE, in a 6-cycle focused laser pulse. Color coded is log_10_[d*ε*_*R*_/dΩ] rad^−2^, with the emission solid angle Ω. The laser and electron beam counterpropagate, with the initial propagation polar angles ${\theta }_{L}^{(i)}=0^\circ$ and ${\theta }_{e}^{(i)}=180^\circ$, respectively. All other parameters are given in the text.

A typical angular distribution of radiation which carries the signature of the stochastic nature of photon emission, is illustrated in Fig. 1; *ϕ* = 0° and ±180° correspond to the positive and negative directions of the laser polarization, respectively. The peak intensity of the 6-cycle (FWHM) laser pulse is *I* ≈ 4.9 × 10^23^ W/cm^2^ (*ξ* = 600), *λ*_0_ = 1 *μ*m, and *w*_0_ = 2 *μ*m. The electron beam, with radius *w*_*e*_ = *λ*_0_, length *L*_*e*_ = 6*λ*_0_, and density *n*_*e*_ ≈ 10^15^ cm^−3^, initially counterpropagates with the laser pulse, i.e. ${\theta }_{e}^{(i)}=180^\circ$. The initial mean kinetic energy of the electron beam is *ε*_0_ = 180 MeV (*γ*_0_ ≈ 353, the maximum value of *χ* during interaction ${\chi }_{max}\mathop{ < }\limits_{ \tilde {}}1$), and the energy and angular spread are Δ*ε*/*ε*_0_ = Δ*θ* = 0.02. The electron-beam parameters are typical for current laser-plasma acceleration setups^5–7^.

Figures 1(a,b) demonstrate RAD including and excluding SE, respectively. The radiation is most significant along the strongest field component *E*_*x*_ of the linearly polarized laser field. However, in a tightly focused laser beam, other components of the electric field, *E*_*y*_ and *E*_*z*_, are not negligible and play a significant role in the electron dynamics and the photon emission. Consequently, the electrons radiate continuously over the azimuthal angle *ϕ*, with a Gaussian radiation distribution with respect to *ϕ* corresponding to the laser transverse profile. Moreover, the radiation sweeps from the polar angle $\theta =180^\circ ={\theta }_{e}^{(i)}$ down to *θ* ≈ 11°. The electrons initially counterpropagate with the laser pulse, emit forwards, and create a high-intensity spectral region around *θ* ≈ 180°. Due to radiative losses, the electron energy decreases, facilitating the reflection condition *γ* ≈ *ξ*/2 in the strong-field region and inducing electron reflection, during which the radiation flash sweeps down to *θ* ≈ 11°. The emission angle after the reflection with respect to the electron average motion $\delta \theta \sim \xi /\gamma$ is determined by the values of *ξ* and *γ in situ* after the reflection. In the region of *θ* ≈ 0°, the radiation intensity is vanishing, because of the very low *χ* value for the co-propagating electron: the radiation energy *ε*_*R*_ ∝ *χ* ∝ *γξ*(1−*β* cos *θ*) ≈ 0 at *θ* ≈ 0°.

The radiation distributions with respect to the polar angle *θ* with and without SE are essentially different in Fig. 1. While with SE the RAD is smoothly peaking at *θ* = 180° and at small angles, without SE it shows a band structure corresponding to the radiation emerging from different laser cycles. A similar behaviour is seen in RAD for photon numbers (See the Supplemental Materials for the details, which includes the impacts of different parameters on the radiation angular distributions).

**Figure 2:**
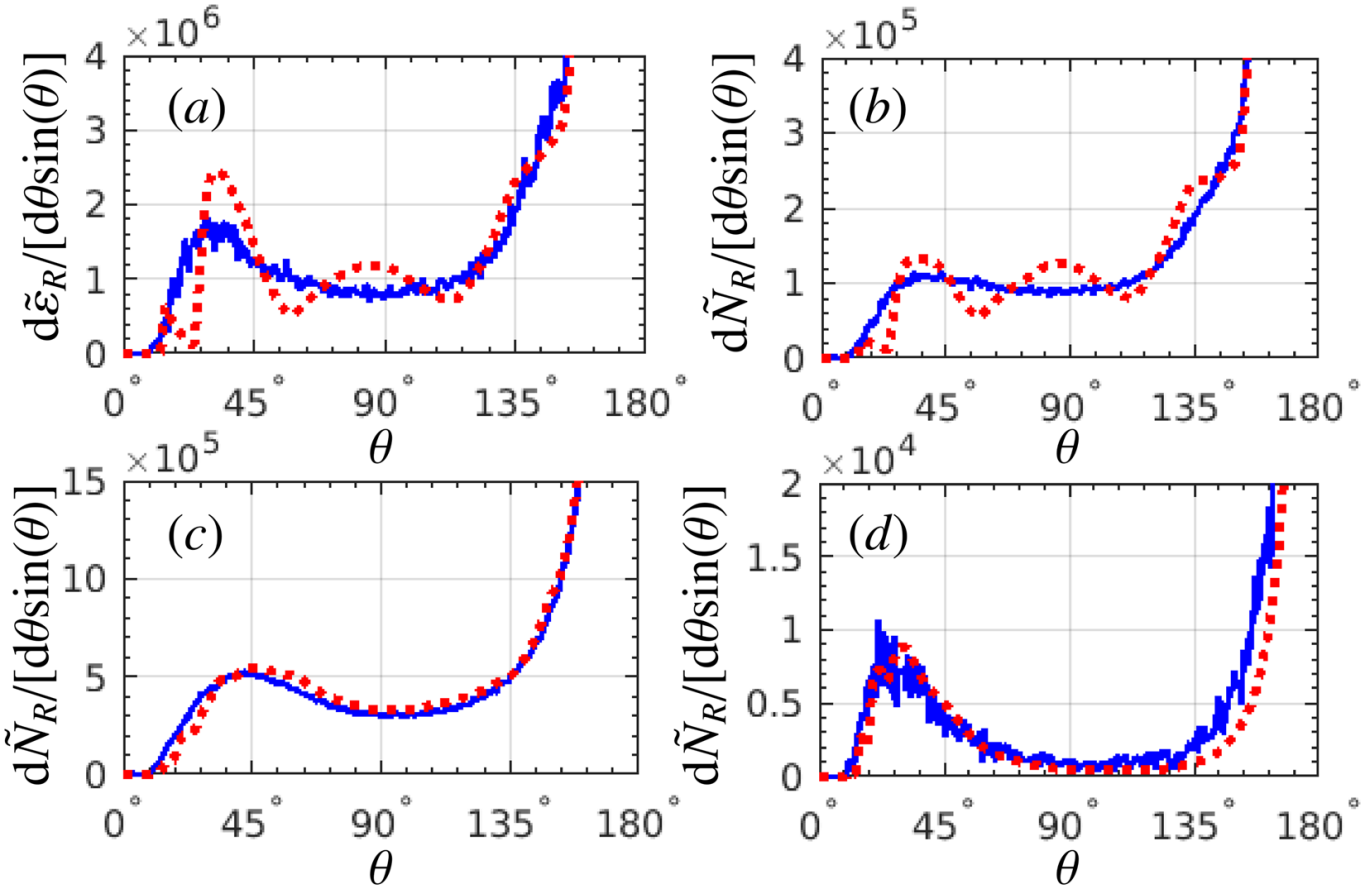
RAD: (**a**) radiation energy d${\tilde{\varepsilon }}_{R}$/[d*θ* sin(*θ*)] (rad^−1^), and (**b**) photon number d${\tilde{N}}_{R}$/[d*θ* sin(*θ*)] (rad^−1^), emitted in the region of −15° ≤ *ϕ* ≤ +15° of the azimuthal angle, see Fig. 1. The emitted photon number d${\tilde{N}}_{R}$/[d*θ* sin(*θ*)] in the whole 2*π*-region of the azimuthal angle: (**c**) all photon energies, and (**d**) the photon energies above 50 MeV. Blue-solid curves are with SE, and red-dotted curves are without SE. The employed laser and electron beam parameters are the same as in Fig. 1.

A quantitative comparison between RADs including and excluding SE is represented in Fig. 2. We focus on the strongest radiation domain along the polarization plane in the region of −15° ≤ *ϕ* ≤ +15°, analysing the radiation energy d${\tilde{\varepsilon }}_{R}$/[d*θ* sin(*θ*)] = ${\int }_{-{15}^{\circ }}^{+{15}^{\circ }}$ d*ϕ* d*ε*_*R*_/dΩ and the photon number d${\tilde{N}}_{R}$/[d*θ* sin(*θ*)] = ${\int }_{-{15}^{\circ }}^{+{15}^{\circ }}$ d*ϕ* d*N*_*R*_/dΩ in this domain. The stochastic nature of photon emission is clearly discernible in RAD: a single broad high-intensity gamma-photon peak is formed in the near-reflection direction when SE is included, while in the case without SE multiple radiation peaks emerge corresponding to the emission from different laser cycles. Therefore, the main detectable difference of RADs with and without SE is the peak number of the radiation: the previous has only one radiation peak, and the latter has several peaks corresponding to the laser-cycle structure.

## Analysis of the radiation properties

**Figure 3:**
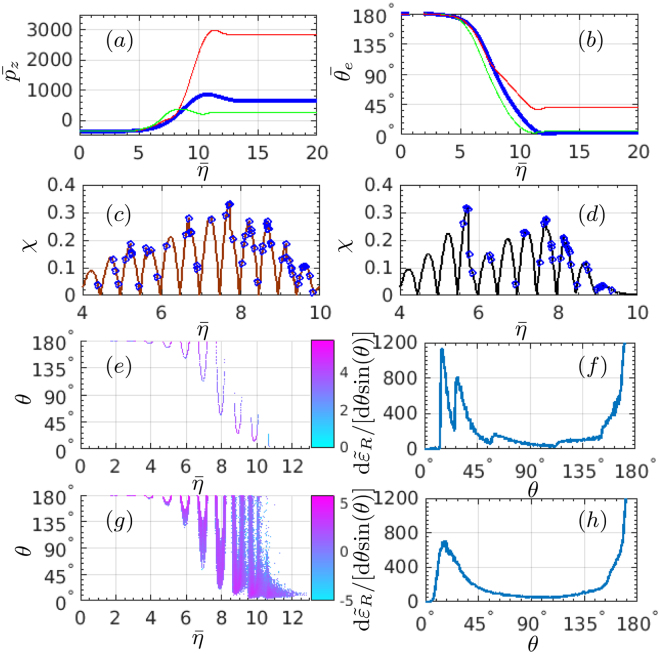
(**a**) The mean longitudinal momentum ${\bar{p}}_{z}$ and (**b**) the mean motion direction (photon-emission direction) ${\bar{\theta }}_{e}$ (polar angle) of a sample electron. The thick line is without SE. The two thin lines are with SE and show the boundaries at $\bar{\eta }\mathop{ > }\limits_{ \tilde {}}10$ (note that the same color lines in (**a**,**b**) do not correspond to the same electron). (**c**,**d**) The variation of the parameter *χ* with respect to $\bar{\eta }$ of a sample electron with SE. The blue marks indicate the photon emissions. The single electron radiation, integrated over the azimuthal angle of −15° ≤ *ϕ* ≤ +15°, without SE: (**e**) radiation intensity vs emission phase $\bar{\eta }$, log_10_[d${}^{2}\tilde{\varepsilon }_{R}$/[d$\bar{\eta }$d*θ* sin(*θ*)]], and (**f**) radiation energy d${\tilde{\varepsilon }}_{R}$/[d*θ* sin(*θ*)]. (**g**,**h**) Correspond to (**e**,**f**), respectively, for the case including SE. The sample electron parameters are given in the text, and the laser parameters are the same as in Fig. 1.

We proceed discussing the role of SE in shaping RAD. The dynamics and radiation of a sample electron is analysed in Fig. 3 (an electron at the beam center, *z* = *L*_*e*_/2 and *x* = *y* = 0, is considered). We follow the mean longitudinal momentum ${\bar{p}}_{z}$ and the mean motion direction ${\bar{\theta }}_{e}$ of the electron during interaction with the laser pulse in dependence on the laser phase $\bar{\eta }=({\omega }_{0}t-{k}_{0}z\mathrm{)/(2}\pi )$, see Fig. 3(a,b), when SE are included or excluded, respectively. To reproduce SE we repeat the simulation 200 times for the same initial conditions of the electron. The single-electron radiation angle is ${\bar{\theta }}_{e}\sim \arctan (m\xi /{\bar{p}}_{z})$ ($0\le {\bar{\theta }}_{e}\le \pi$). The point ${\bar{p}}_{z}=0$ corresponds to the electron reflection, when the emission angle sharply changes from the forward into the backward direction.

In the case without SE, the single-electron radiation angle is well defined at each moment during interaction. In contrast, in the case with SE one observes spreading of the average longitudinal momenta and the emission angles at a certain laser phase $\bar{\eta }$, see Fig. 3(a,b), which stems from the probabilistic character of the photon-emission process. In each possible trajectory, the electron emits photons at different moments and photons of different energies. Consequently, the electron has different energy and emission angle at the same laser phase $\bar{\eta }$ (same *ξ*).

The dynamics of the photon emission in the SE case described above is illustrated in Fig. 3(c,d) for two sample trajectories, having a small and a large energy after the reflection in Fig. 3(a). Although both of the trajectories begin with the same initial condition, in the first case the number of photon emissions before the reflection ($\bar{\eta }\mathop{ < }\limits_{ \tilde {}}7.5$) is larger. In the second case the electron “straggles” (does not emit large number of photons) most of the time before the reflection, only emitting a large photon near the reflection $\bar{\eta }\approx 5.5$ (See the Supplemental Materials for the details, which includes the impacts of different parameters on the radiation angular distributions). Because of the latter, the parameter *χ* after the reflection is larger for the first trajectory with respect to the second, yielding significantly more radiative loss for the first trajectory than for the second one, and correspondingly, smaller energy (larger emissison angle) for the first trajectory than for the second one after the reflection (See the Supplemental Materials for the details, which includes the impacts of different parameters on the radiation angular distributions). Thus, different probabilistic dynamics of the photon emission, i.e. SE, induces spreading of the photon emission angle at each laser phase.

The angle-resolved radiation intensity and radiation energy are shown in Fig. 3(e,f), respectively, for the case without SE. In each cycle strongest radiation arises near the peaks of the cycles at a certain angle. Between adjacent radiation peaks, there is a gap in emission angle corresponding to the weak-field part of the laser cycle. Therefore, the RAD reveals the laser-cycle structure when SE are neglected.

The RAD with SE for a single electron is shown in Fig. 3(g,h). Since with SE the radiation angle in each laser cycle has a very broad spread shown in Fig. 3(b,g), the gaps in emission angle between adjacent radiation peaks of each cycle are filled out. Consequently, the radiation intensity in this case shows a single gamma-radiation peak corresponding to the peak of the laser pulse. Note that the discussed qualitative features of the RAD for a sample electron do not depend on the initial position of the electron in the electron beam. The variation of the initial position introduces only a slight modification of a quantitative character (See the Supplemental Materials for the details, which includes the impacts of different parameters on the radiation angular distributions).

The electron straggling effect in our setup is estimated in Fig. 2(c,d) for the emitted photon number integrated over the whole 2*π*-region of the azimuthal angle. The straggling effect is observed as an enhancement of the emission of high-energy photons when the SE are included, see Fig. 2(d) for the emitted photon number above 50 MeV. However, the electron straggling effect in the considered regime is insignificant and not relevant for the observation of SE.

## Optimal conditions for observation of stochasticity effects

**Figure 4:**
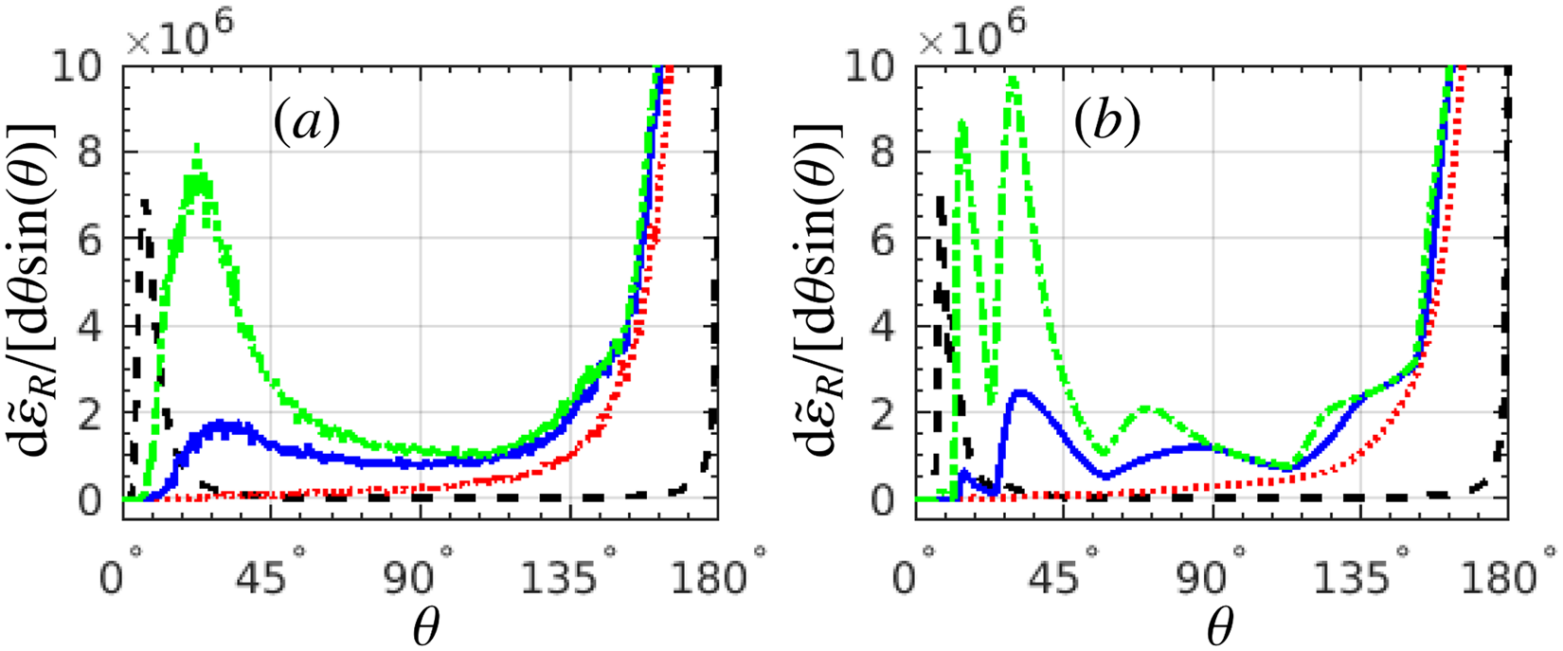
RAD d${\tilde{\varepsilon }}_{R}$/[d*θ* sin(*θ*)]: (**a**) with SE, and (**b**) without SE, respectively. The laser focal radius *w*_0_ equals: (red, dotted) *λ*_0_, (blue, solid) 2*λ*_0_ and (green, dash-dotted) 3*λ*_0_. The black-dash curves show the case of a 6-cycle plane-wave laser pulse, scaled by a factor of 10^−2^. *ξ* = 600 for all cases. All other laser and electron parameters are the same as in Fig. 1.

We investigate the optimization of the laser (focal radius, pulse duration) and electron (energy) parameters for the best identification of SE. The influence of the laser focusing effect on RAD is studied in Fig. 4. The case of *w*_0_ = 2*λ*_0_ is optimal, being the same as in Figs 1 and 2, when the SE are exhibited by a broad smooth peak of the gamma radiation in backward direction. For *w*_0_ = *λ*_0_ = *w*_*e*_, the near-reflection radiation is rather weak, and there is no laser-cycle structure in the “without SE” case. For *w*_0_ = 3*λ*_0_, the main (highest) gamma-photon peaks in the region of $11^\circ \mathop{ < }\limits_{ \tilde {}}\theta \mathop{ < }\limits_{ \tilde {}}45^\circ$ are much stronger apparently than those for *w*_0_ = 2*λ*_0_, since all electrons are much closer to the laser-intensity center, which also weakens the shift of the main peaks in polar angle when the SE are excluded. Consequently, higher angular resolution is required to distinguish the “without SE” case from the one with SE. In the extreme case of a plane-wave laser pulse the structure of multiple gamma-photon peaks in the “without SE” case cannot be observed any more. Therefore, a tightly focused laser pulse with a focal radius of roughly 2 times of the electron-beam radius applies well for the observation of SE.

**Figure 5:**
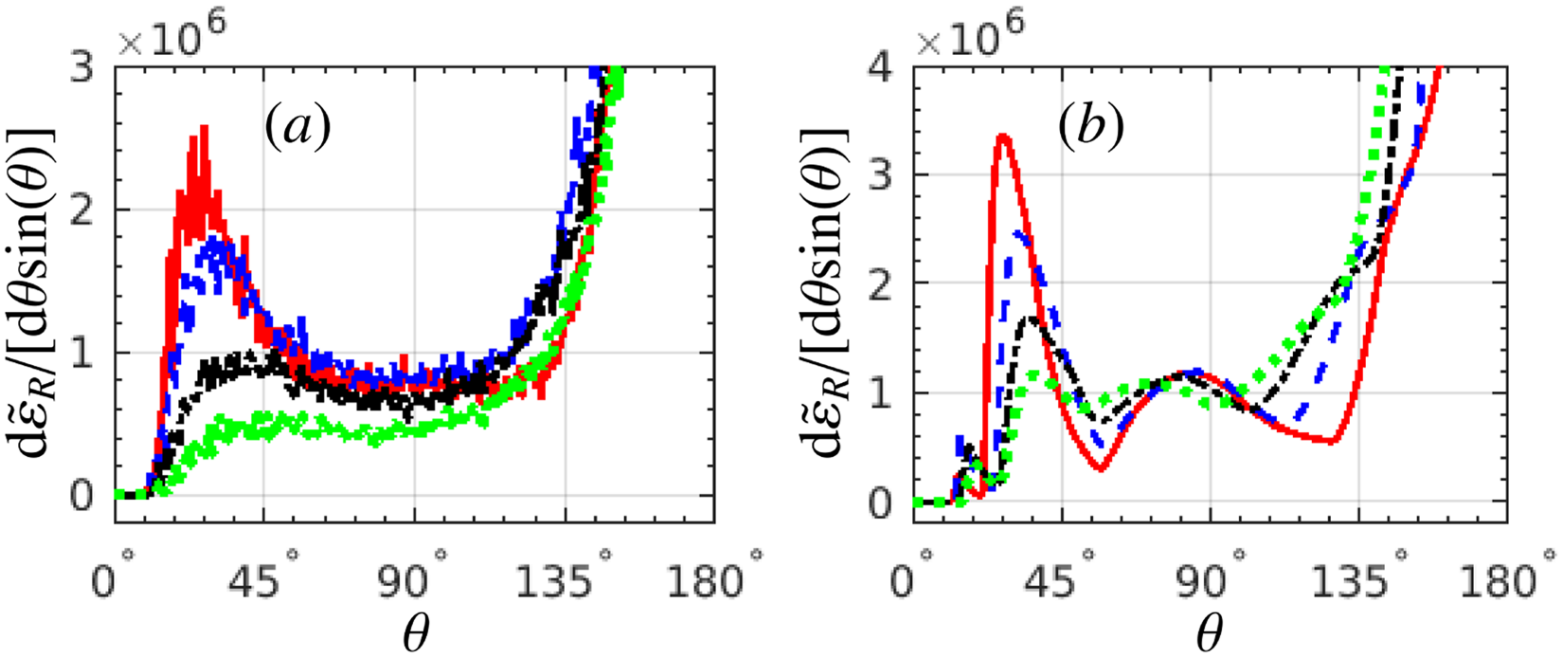
RAD d${\tilde{\varepsilon }}_{R}$/[d*θ* sin(*θ*)]: (**a**) with SE, and (**b**) without SE, respectively. The laser-pulse length *L*_*L*_ is: (red-solid) 4*λ*_0_, (blue-dash) 6*λ*_0_, (black-dash-dotted) 8*λ*_0_ and (green-dotted) 10*λ*_0_. The electron-beam length *L*_*e*_ is chosen to be equal to the corresponding laser-pulse length. *ξ* = 600 for all cases. All other laser and electron parameters are the same as in Fig. 1.

The dependence of RAD on the laser-pulse length is shown in Fig. 5. As the laser-pulse length increases from 4*λ*_0_ to 10*λ*_0_, the main (highest) radiation peak in the region of $11^\circ \mathop{ < }\limits_{ \tilde {}}\theta \mathop{ < }\limits_{ \tilde {}}45^\circ$ gradually decreases, and the plateaus between the peaks gradually increase. In principle, for a longer laser pulse there are more peaks corresponding to the laser cycles in the “without SE” case, which is helpful to distinguish SE. However, for a longer laser pulse, e.g., *L*_*L*_ = 10*λ*_0_, the laser-field gradient is smaller than for shorter laser pulses, consequently, the ratio between the peaks and the plateaus becomes smaller, and the visibility of the signatures becomes worse. Then, a short laser pulse of *L*_*L*_ ≈ 6*λ*_0_ is optimal: it has a more-cycle structure than for ultrashort pulses, and the visibility of the laser-cycle structure is better than longer pulses.

**Figure 6:**
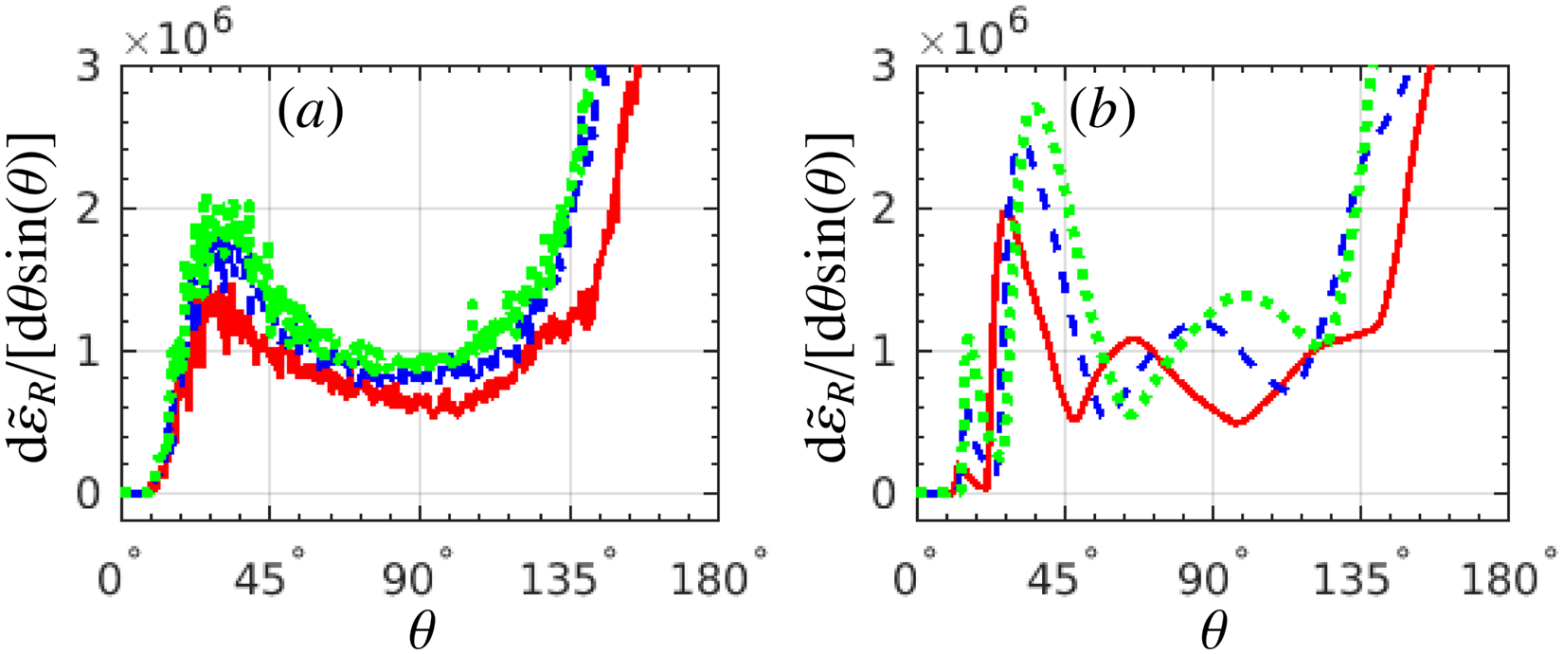
RAD d${\tilde{\varepsilon }}_{R}$/[d*θ* sin(*θ*)]: (**a**) with SE, and (**b**) without SE, respectively. The mean kinetic energy of the electron beam *ε*_0_ is: (red-solid) 120 MeV, (blue-dashed) 180 MeV and (green-dotted) 240 MeV, respectively. All other laser and electron parameters are the same as in Fig. 1.

The role of the initial kinetic energy of the electron beam on the radiation angular distribution is analysed in Fig. 6. As the electron kinetic energy *ε*_0_ increases from 120 MeV to 240 MeV, the radiation energy *ε*_*R*_ ∝ *χ* ∝ *ξγ* increases as well since $\gamma \sim {\varepsilon }_{0}$ and *ξ* being constant is assumed. The peaks in RAD shift right to a larger polar angle since the emission angle after reflection satisfies $\delta \theta =({\theta }_{e}^{(i)}-{\theta }_{e})\sim \xi /\gamma$. Therefore, larger initial kinetic energies are favourable for generation of more clear SE signatures, but one has to keep in mind that for the discussed SE features the reflection condition $\gamma \sim \xi \mathrm{/2}$ should be held in advance.

**Figure 7:**
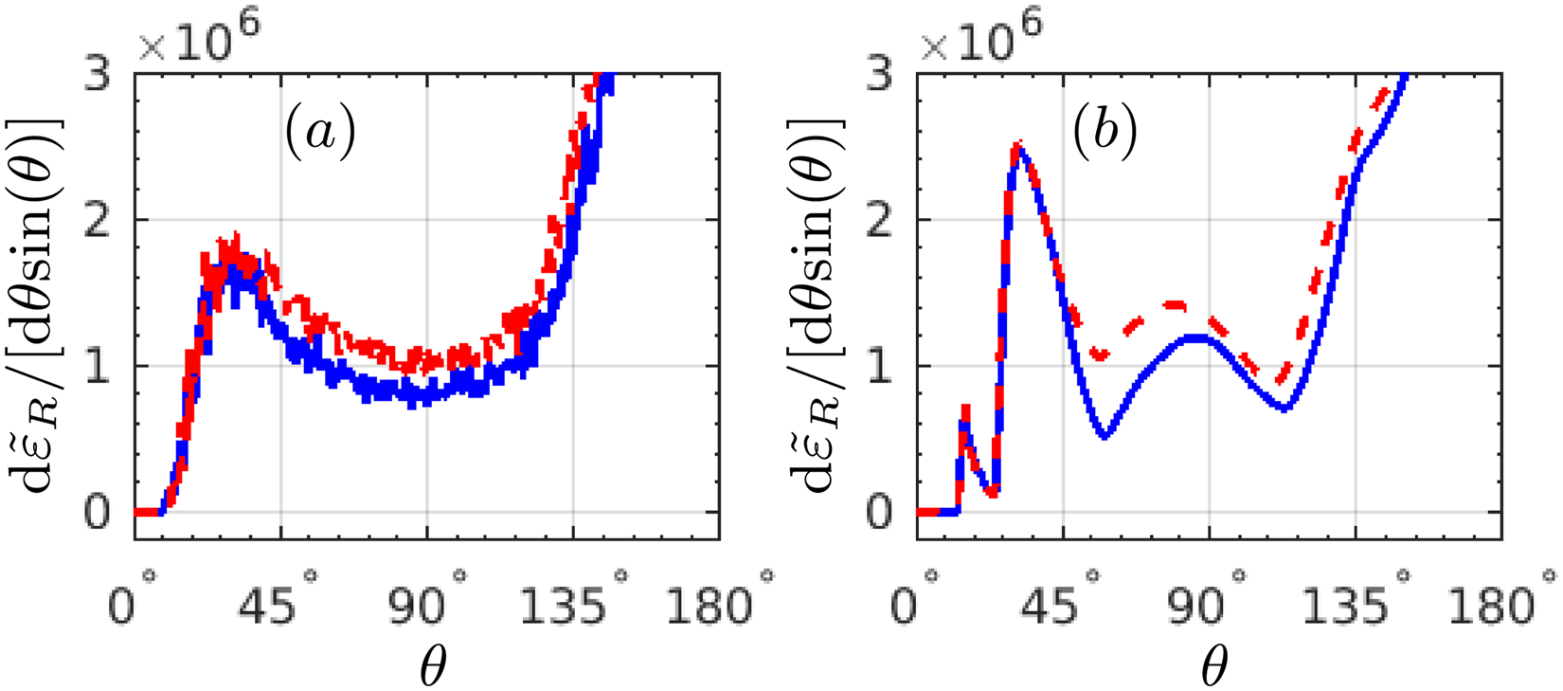
RAD d${\tilde{\varepsilon }}_{R}$/[d*θ* sin(*θ*)]: (**a**) with SE, and (**b**) without SE, respectively. The blue-solid and red-dash curves present the results with *θ*_colliding_ = 180° and 175°, respectively. All other laser and electron parameters are the same as in Fig. 1.

Finally, the role of the colliding angle between the laser pulse and the electron beam is investigated, as shown in Fig. 7. The figure shows that the variation of the colliding angle of 5° does not change the qualitative SE signatures. This is the strength of the qualitative SE signatures that they are robust with respect to the variation of the process parameters.

## Conclusion

We have revealed signatures of the stochastic nature of gamma-photon emission during nonlinear Compton scattering of a superstrong short focused laser pulse by a counterpropagating electron beam in the quantum radiation-dominated regime. The signatures are manifested in the qualitative features of the angular distribution of the radiation in the near-backward direction with respect to the initial electron motion, which arises when the electron energy is at the reflection condition. When the stochasticity effects are included, a single broad gamma-photon peak is formed in the near-reflection radiation angular distribution, while several gamma-photon peaks would arise if there were no stochasticity in the photon emission. The signatures are enhanced with tightly focused and short laser pulses. They are robust with respect to variation of the laser and electron parameters and can be observed in near-future laser facilities, such as ELI and XCELS.

## Methods

In this work, we employ a linearly polarized focused Gaussian laser pulse propagating along +*z* direction and polarized in *x* direction. The expressions of the electromagnetic fields are presented in the following refs 46 and 47:

1 $${E}_{x}=-iE\,[1+{\varepsilon }^{2}({f}^{2}{\tilde{x}}^{2}-\frac{{f}^{3}{\rho }^{4}}{4})],$$

2 $${E}_{y}=-iE{\varepsilon }^{2}{f}^{2}\tilde{x}\tilde{y},$$

3 $${E}_{z}=E\,[\varepsilon f\,\tilde{x}+{\varepsilon }^{3}\tilde{x}\,(-\frac{{f}^{2}}{2}+{f}^{3}{\rho }^{2}-\frac{{f}^{4}{\rho }^{4}}{4})],$$

4 $${B}_{x}=\mathrm{0,}$$

5 $${B}_{y}=-iE\,[1+{\varepsilon }^{2}\,(\frac{{f}^{2}{\rho }^{2}}{2}-\frac{{f}^{3}{\rho }^{4}}{4})],$$

6 $${B}_{z}=E\,[\varepsilon f\,\tilde{y}+{\varepsilon }^{3}\tilde{y}(\frac{{f}^{2}}{2}+\frac{{f}^{3}{\rho }^{2}}{2}-\frac{{f}^{4}{\rho }^{4}}{4})],$$

where,

7 $$E={E}_{0}{F}_{n}f{e}^{-f{\rho }^{2}}{e}^{i(\eta +{\varphi }_{{\rm{CEP}}})}{e}^{-\frac{{\tau }^{2}}{{T}_{0}^{2}}},$$

*E*_0_ is the amplitude of the laser fields with normalization factor *F*_*n*_ = *i* to keep $\sqrt{{E}_{x}^{2}+{E}_{y}^{2}+{E}_{z}^{2}}={E}_{0}$ at the focus, yielding the scaled coordinates

8 $$\tilde{x}=\frac{x}{{w}_{0}},\quad \tilde{y}=\frac{y}{{w}_{0}},\quad \tilde{z}=\frac{z}{{z}_{r}},\quad {\rho }^{2}={\tilde{x}}^{2}+{\tilde{y}}^{2},$$

where ${z}_{r}=\frac{{k}_{0}{w}_{0}^{2}}{2}$ is the Rayleigh length, $f=\frac{i}{\tilde{z}+i}$, *η* = *ω*_0_ *t* − *k*_0_ *z*, and *ϕ*_CEP_ the carrier-envelop phase. Note that *ϕ*_CEP_ = 0 is employed throughout.

In superstrong laser fields $\xi \gg 1$, the photon emission probability *W* is determined by the local electron trajectory, consequently, by the local value of the parameter *χ* ^38, 44^:

9 $$\frac{{d}^{2}W}{d\eta d\omega }=\frac{\alpha \chi {m}^{2}[{\int }_{{\omega }_{r}}^{\infty }\,{K}_{\mathrm{5/3}}(x)dx+\omega {\omega }_{r}{\chi }^{2}{K}_{\mathrm{2/3}}({\omega }_{r})]}{\sqrt{3}\pi ({k}_{0}\cdot p)},$$

where $\omega =({k}_{0}\cdot k)/\tilde{\chi }({k}_{0}\cdot p)$ is the normalized emitted photon energy, *k*_0_, *k* and *p* are the four-vectors of the driving laser photon, the emitted photon and the electron, respectively, and *ω*_*r*_ = 2*ω*/3(1 − *χω*). The applicability of the constant crossed-field approximation for the emission probability in Eq. (9) related to the laser intensity has been estimated and justified (See the Supplemental Materials for the details, which includes the impacts of different parameters on the radiation angular distributions). The photon emission of electrons is considered to be a Monte-Carlo stochastic process^40–42^. During the electron-laser interaction, for each propagation coherent length Δ*η*, the photon emission will take place if the condition (*dW*/d*η*)Δ*η* ≥ *N*_*r*_ is fulfilled, where *N*_*r*_ is a uniformly distributed random number in $\mathrm{[0},\,1]$. Herein, the coherent length Δ*η* is inversely proportional to the invariant laser field parameter *ξ*, i.e., ${\rm{\Delta }}\eta \sim \mathrm{1/}\xi$. However, to keep the total photon emission energy consistent, i.e., to exclude numerical error of the simulation of photon emission, we choose ${\rm{\Delta }}\eta \sim \mathrm{1/}\xi$. The photon emission probability

$$W={\rm{\Delta }}\eta \frac{dW}{d\eta }={\rm{\Delta }}\eta {\int }_{{\omega }_{min}}^{{\omega }_{max}}\frac{{d}^{2}W}{d\eta d\omega }d\omega ,$$

where *ω*_*min*_ is the driving laser photon energy, and *ω*_*max*_ equals the electron instantaneously kinetic energy. In addition, the emitted photon energy *ω*_*R*_ is determined by the relation:

$$\frac{1}{W}{\int }_{{\omega }_{min}}^{{\omega }_{R}}\frac{dW(\omega )}{d\omega }d\omega =\frac{{\rm{\Delta }}\eta }{W}{\int }_{{\omega }_{min}}^{{\omega }_{R}}\,\frac{{d}^{2}W(\omega )}{d\eta d\omega }d\omega ={\tilde{N}}_{r},$$

where, ${\tilde{N}}_{r}$ is an another independent uniformly distributed random number in $\mathrm{[0,}\,1]$. Between the photon emissions, the electron dynamics in the laser field is governed by classical equations of motion:

10 $$\frac{d{\boldsymbol{p}}}{dt}=-e({\boldsymbol{E}}+{\boldsymbol{\beta }}\times {\boldsymbol{B}}).$$

Given the smallness of the emission angle ~1/*γ* for an ultrarelativistic electron, the photon emission is assumed to be along the electron velocity^8, 38^. The photon emission induces the electron momentum change ***p***_*f*_ ≈ (1 − *ω*_*R*_/*cp*)***p***_*i*_, where ***p***_*i*,*f*_ are the electron momentum before and after the emission, respectively. In this simulation, the interference effects between emissions in adjacent coherent lengths are negligible since the laser fields employed are superstrong, i.e., $\xi \gg 1$. Therefore, the photon emissions happening in each coherent length are independent of each other. However, as the interference effects are significant, i.e. $\xi \mathop{ < }\limits_{ \tilde {}}1$, the photon emission probabilities in adjacent coherent lengths are summed up to compare with the random number *N*_*r*_ until the photon emission happens.

As the stochastic effects are not taken into account, the rate of the electron radiation loss ${\mathcal I}$ can be considered as

11 $$ {\mathcal I} ={\int }_{{\omega }_{min}}^{{\omega }_{max}}\,d\omega ({k}_{0}\cdot k)\frac{{d}^{2}W}{d\eta d\omega }.$$

Implementing the radiation losses due to quantum radiation reaction into the electron classical dynamics leads to the following equation of motion^22–24^:

12 $$\frac{d{p}^{\alpha }}{d\tilde{\tau }}=\frac{e}{m}{F}^{\alpha \beta }{p}_{\beta }-\frac{ {\mathcal I} }{m}{p}^{\alpha }+{\tau }_{c}\frac{ {\mathcal I} }{{ {\mathcal I} }_{c}}{F}^{\alpha \beta }{F}_{\beta \gamma }{p}^{\gamma },$$

where $\tilde{\tau }$ is the proper time,

13 $$\frac{d}{d\tilde{\tau }}=({k}_{0}\cdot p)\frac{d}{d\tilde{\xi }},\quad \tilde{\xi }=({k}_{0}\cdot \tilde{x})$$

$\tilde{x}$ the four-vector of the coordinate, *τ*_*c*_ ≡ 2*e* ^2^/(3*m*), and ${ {\mathcal I} }_{c}=2\alpha {\omega }^{2}{\xi }^{2}$ the classical radiation loss rate. The radiation energy and photon number at each step from the initial phase *η*_*i*_ to the final phase *η*_*f*_ read:

14 $${\varepsilon }_{R}={\int }_{{\eta }_{i}}^{{\eta }_{f}}\, {\mathcal I} d\eta ,\quad {N}_{R}=W.$$

Then, the photon number of the radiation energy *ε*_*R*_ above an arbitrary energy *ε*_*arb*_ at each step yields:

15 $${W}_{arb}={\int }_{{\omega }_{arb}}^{{\omega }_{max}}\,d\omega \,{\int }_{{\eta }_{i}}^{{\eta }_{f}}\,d\eta \frac{{d}^{2}W}{d\eta d\omega },$$

where *ω*_*arb*_ = *ε*_*arb*_.

**Figure 8:**
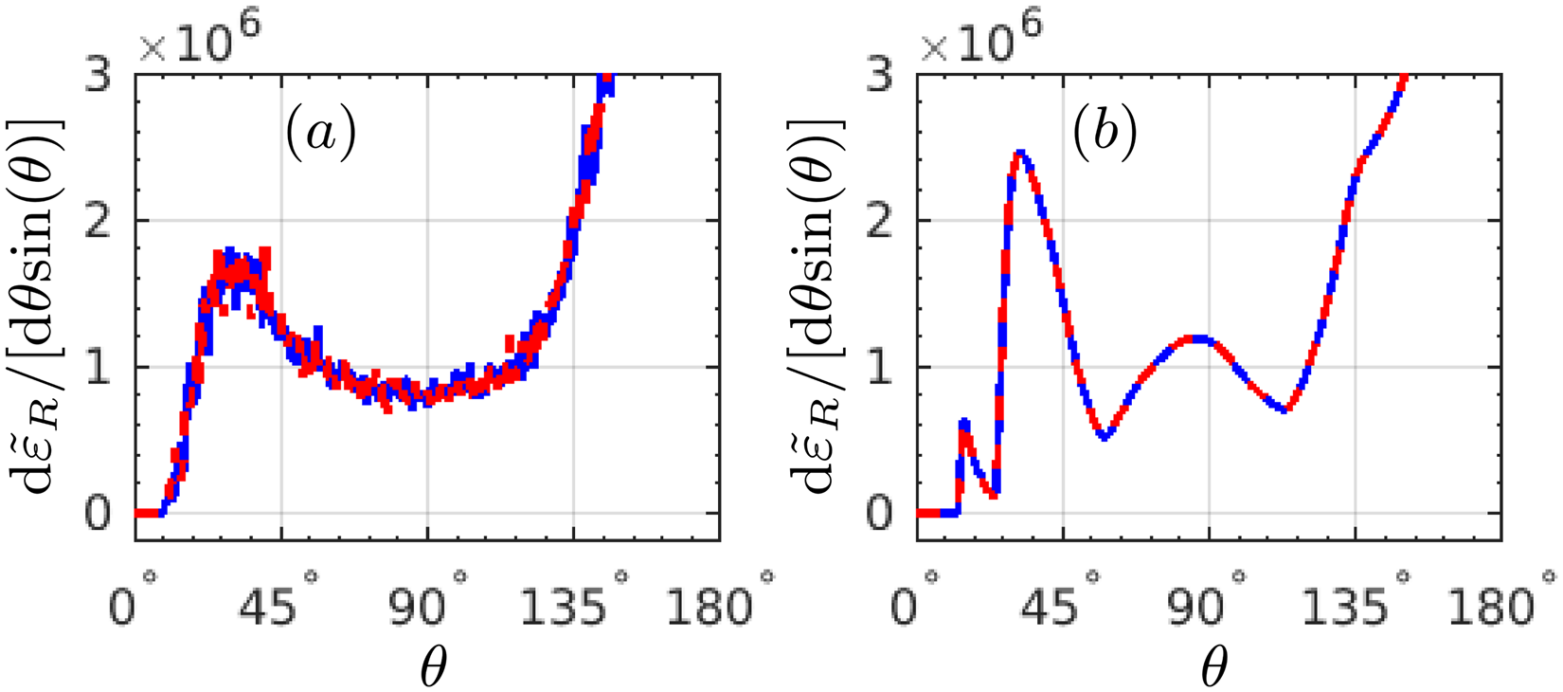
RAD d${\tilde{\varepsilon }}_{R}$/[d*θ* sin(*θ*)]: (**a**) with SE, and (**b**) without SE, respectively. The blue-solid and red-dash curves present the results with the simulation step number *N*_step_ = 2.7 × 10^4^ and 5.4 × 10^4^. All other laser and electron parameters are the same as in Fig. 1.

To prove explicitly the convergence of our Monte-Carlo simulations, we re-simulate the radiation of Fig. 2 via employing a smaller step size, as shown in Fig. 8. The blue-solid curves present the same radiation as in Fig. 2 with the simulation step number *N*_step_ = 2.7 × 10^4^, and the red-dash curves show the radiation with a smaller simulation step size, i.e., larger step number of *N*_step_ = 5.4 × 10^4^. The simulations clearly show that the behaviour of the photon emission angular distribution does not change with decreasing the simulation step size, i.e., increasing the simulation step number, which indicates the convergence of our results. Besides, the simulations based on the “Sokolov” equation have been compared with those based on a modified “Landau-Lifshitz” equation (rescaled radiation but no stochasticity effects)^48^, and they demonstrate good coincidences (See the Supplemental Materials for the details, which includes the impacts of different parameters on the radiation angular distributions).

## Electronic supplementary material

Supplemental materials
