# Supplementary material for: Angle-resolved stochastic photon emission in the quantum radiation-dominated regime
Source: Sci Rep. 2017 Sep 14;7:11556. doi: 10.1038/s41598-017-11871-0 (PMC5599677; doi:10.1038/s41598-017-11871-0)
Supplement: Supplementary file 1 — Supplemental materials [file 41598_2017_11871_MOESM1_ESM.pdf]

Supplemental materials
# of the article "Angle-resolved stochastic photon emission in the quantum radiation-dominated regime"

Jian-Xing Li, Yue-Yue Chen, Karen Z. Hatsagortsyan, and Christoph H. Keitel

I. THE RADIATED PHOTON NUMBER

Figure 1 represents the angular distribution of the radiated photon number $\mathrm{d}N_R/\mathrm{d}\Omega$, corresponding to Fig. 1 of the paper, which shows the radiated energy $\mathrm{d}\varepsilon_R/\mathrm{d}\Omega$. We employ the same electron and laser parameters as in Fig. 1 of the paper: the electron beam counterpropagates with a 6-cycle (FWHM) laser pulse with a peak intensity $I \approx 4.9\times 10^{23}\mathrm{W/cm}^2$ ($\xi = 600$), and the initial polar angle of the electron motion is $\theta_e^{(i)} = 180°$. The electron-beam radius is $w_e = 1\mu$m, the electron-beam length equals the laser-pulse length, $L_e = L_L = 6\lambda_0$, the electron density $n_e \approx 1.27\times 10^{15}$ cm^{-3}, and the initial electrons are randomly distributed in the bunch. The initial mean kinetic energy of the electron beam is $\varepsilon_0 = 180$ MeV ($\gamma_0 \approx 353.25$ and $\chi \lesssim 0.5$), the energy and angular spread of the electron beam are $\Delta\varepsilon/\varepsilon_0 = \Delta\theta = 0.02$, and they both have a Gaussian distribution. The distributions of $\mathrm{d}N_R/\mathrm{d}\Omega$ including and excluding the stochasticity effects (SE) represent similar structures as those of $\mathrm{d}\varepsilon_R/\mathrm{d}\Omega$ in Fig. 1 of the paper.

For observation of the predicted SE the angular resolution of gamma-ray detection should be less than 10°, with the precision of the photon number detection less than 10^3 photons, which is within the commonly achieved resolution.

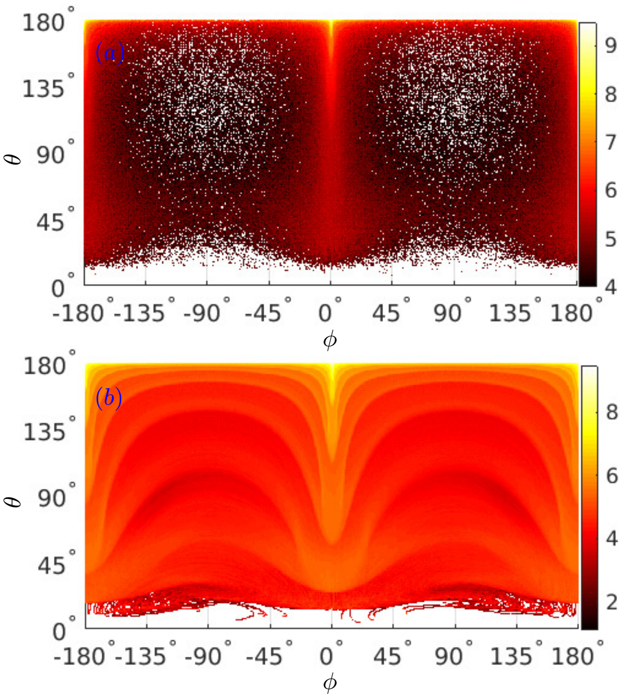

FIG. 1. The radiation's angular distribution: (a) with SE, and (b) without SE. Color coded is $\log_{10}[\mathrm{d}N_R/\mathrm{d}\Omega]$ rad^{-2} in the polar angle θ and the azimuthal angle ϕ, with the radiation photon number N_R and the emission solid angle Ω. $\lambda_0 = 1\mu$m, and the laser focal radius is $w_0 = 2\mu$m. Other laser and electron beam parameters are given in the text of the article.

II. APPLICABILITY OF THE CONSTANT CROSSED-FIELD APPROXIMATION FOR THE EMISSION PROBABILITY

To estimate the range of applicability of the constant crossed-field approximation for the emission probability, let us return to the original work of V. I. Ritus, where the radiation probability is calculated in a plane laser field without any approximation, see Ref. 38. In the limit $\xi \gg 1$ the asymptotic formula for the generalized Bessel function, which describes the emission probability, is derived coinciding with the crossed-field result. The applied approximation is to neglect in the exponent derivative terms higher than the third derivative, see Eq. (2) of Appendix B of Ref. 38.

Let us analyze the neglected terms in the derivation of the asymptotic formula, which will provide us with an estimate of the order of magnitude of possible deviations from the exact formula. The derivation using the saddle-point integration of the general formula $A_0(s,\alpha,\beta) \sim \mathrm{Re}\frac{1}{\pi}\int_0^\pi e^{f(\phi)}d\phi$ is given in Appendix B, where $\alpha = eap/kp$, $\beta = e^2a^2/8kp$, s is integer, e the electron charge, k the four-vector of the laser wave, p a constant four-vector that describes the state of the particle in the field of the wave and coincides with the particle momentum when the field is turned off, and a the amplitudes of the potential. The function in the exponent is expanded near the saddle-point:

$f(\phi) \approx f(\phi_0)+\frac{1}{2!}f''(\phi_0)(\phi-\phi_0)^2+\frac{1}{3!}f'''(\phi_0)(\phi-\phi_0)^3+\frac{1}{4!}f''''(\phi_0)(\phi-\phi_0)^4+\ldots$.

It is shown that $f''(\phi_0)\sim s^{2/3}$, $f'''(\phi_0)\sim f''''(\phi_0)\sim s$, and higher order derivatives are also $\sim s$ at $s = s_0$. The second derivative term determines the saddle-point integration region $(\phi-\phi_0)\sim 1/s^{1/3}$ from the condition $\frac{1}{2!}f''(\phi_0)(\phi-\phi_0)^2\sim 1$. The third derivative term is of the order of $f'''(\phi_0)(\phi-\phi_0)^3\sim 1$, with $(\phi-\phi_0)\sim 1/s^{1/3}$, and therefore, both terms are kept in the exponent, while higher order terms are neglected. The largest one is the forth derivative term $f''''(\phi_0)(\phi-\phi_0)^4\sim s(1/s^{1/3})^4\sim s^{1/3}$. Thus, the neglected term in the exponent is of the order of $s^{1/3}\sim 1/\xi$, because the effective number of the

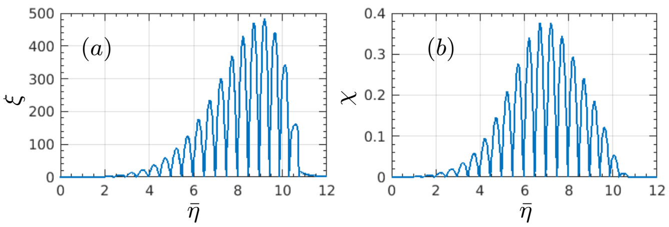

FIG. 2. The variations of (a) the invariant laser field parameter ξ and (b) the invariant parameter χ with respect to the laser phase $\bar{\eta} = \eta/(2\pi)$ without SE. The laser and electron parameters are given in the text.

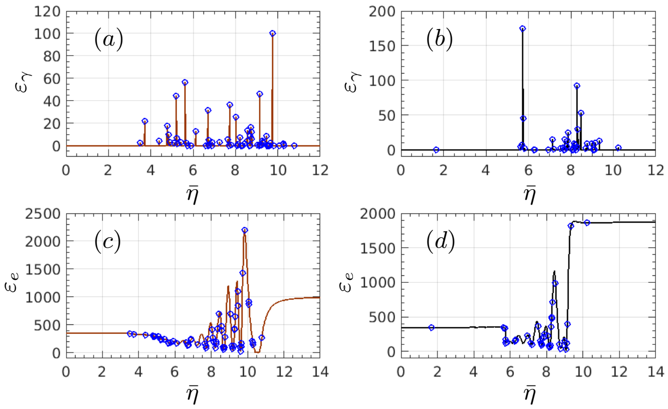

FIG. 3. (a) and (b): the variation of the photon energy radiated by the electron ε_γ with respect to the laser phase $\bar{\eta}$. (c) and (d): the variation of the electron energy ε_e with respect to $\bar{\eta}$. (a) and (c) correspond to Fig. 3(c) of the paper, and (b) and (d) correspond to Fig. 3(d) of the paper. The SE are included in this figure.

absorbed laser photons is $s \sim \xi^3$. Then, the neglected terms in the probability is of the order of $1/\xi$, which means that at $\xi \sim 10$, the accuracy of the synchrotron formula (crossed-field approximation) is 90%, i.e., the relative error is of 10%.

III. ON THE EXPLANATION OF THE EMISSION ANGLE SPREADING

As shown in Figs. 3(a) and 3(b) of the paper, in the case without SE, the single-electron radiation angle $\bar{\theta}_e \sim \arctan(m\xi/\bar{p_z})$ $(0 \leq \bar{\theta}_e \leq \pi)$ is well defined at each moment during interaction. The radiation is assumed to be along the electron motion since the radiation-angle spread of order $1/\gamma$ is rather small. The electron initially counterpropagating with the laser pulse has a negative $\bar{p_z}$, which decreases by an absolute value during the interaction due to radiative energy loss, while the laser field ξ increases in subsequent cycles, and the emission angle decreases. The point $\bar{p_z} = 0$ corresponds to the electron reflection, when the emission angle sharply changes from the forward into the backward direction. After reflection

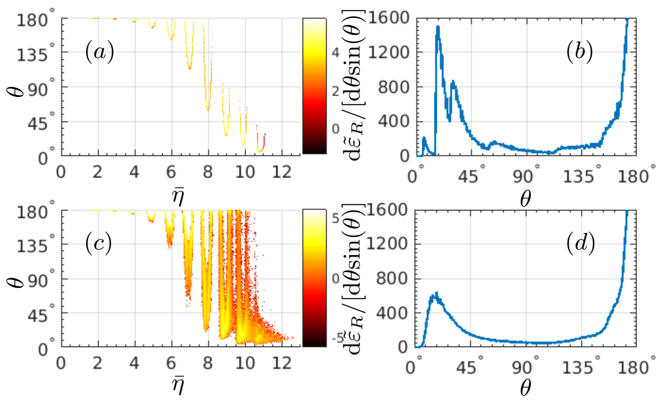

FIG. 4. The radiation's angular distribution excluding SE: (a) radiation intensity $\log_{10}[\mathrm{d}^2\tilde{\varepsilon}_R/[\mathrm{d}\bar{\eta}\mathrm{d}\theta\sin(\theta)]]$ rad^{-1}, and (b) radiation energy $\mathrm{d}\tilde{\varepsilon}_R/[\mathrm{d}\theta\sin(\theta)]$ rad^{-1}, integrated over the azimuthal angle in the region of $-15^\circ \leq \phi \leq +15^\circ$. (c) and (d) are corresponding to (a) and (b), respectively, for the case with SE. The initial coordinates of the sample electron are $z = x = y = 0$, and other electron and laser parameters are the same as in Fig. 1.

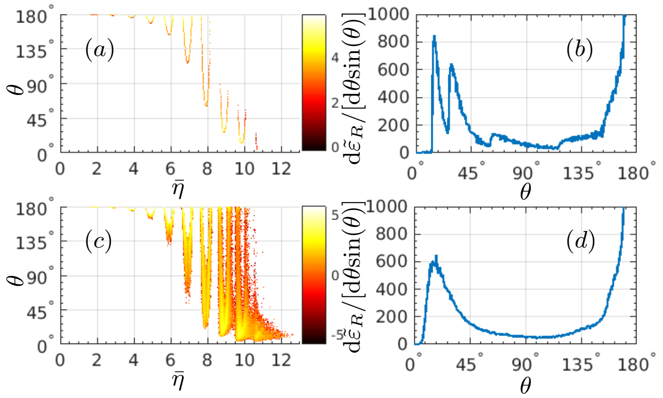

FIG. 5. The radiation's angular distribution excluding SE: (a) radiation intensity $\log_{10}[\mathrm{d}^2\tilde{\varepsilon}_R/[\mathrm{d}\bar{\eta}\mathrm{d}\theta\sin(\theta)]]$ rad^{-1}, and (b) radiation energy $\mathrm{d}\tilde{\varepsilon}_R/[\mathrm{d}\theta\sin(\theta)]$ rad^{-1}, integrated over the azimuthal angle in the region of $-15^\circ \leq \phi \leq +15^\circ$. (c) and (d) are corresponding to (a) and (b), respectively, for the case including the SE. The initial coordinates of the sample electron are $z = L_e$ and $x = y = 0$, and other electron and laser parameters are the same as in Fig. 1.

$\bar{\eta} \gtrsim 7.5$ $(\bar{p_z} > 0)$, $\bar{\theta}_e$ further decreases because of an increase of $\bar{p_z}$ when the electron is accelerated along the laser pulse propagation direction.

The angle-resolved radiation intensity and radiation energy are shown in Figs. 3 (e) and 3(f) of the paper, respectively, for the case without SE. In each cycle the smallest θ_e corresponds to the peak of the field (the largest ξ). The strong backward radiation arises near the peaks of the cycles around $\bar{\eta} = 8$, 9 and 10, respectively, after the reflection point when ξ and χ are both large, creating peaks at $\theta \approx 61^\circ$, 28° and 16°, respectively, in Fig. 3 (f) of the paper. The radiation at $\bar{\eta} > 10.5$ is weak

since $\varepsilon_R \sim \chi$ is rather small at $\theta \approx 0°$. Between adjacent radiation peaks, there is a gap corresponding to the weak-field part of the laser cycle. Therefore, the radiation angular distribution (RAD) reveals the laser-cycle structure when SE are neglected.

The RAD with SE for a single electron is shown in Figs. 3(g) and 3(h) of the paper, which are reproduced via simulating $2.4{\times}10^4$ times the electron-radiation process with the same initial conditions. Since with SE the radiation angle in each laser cycle has a very broad spread, the gaps between adjacent radiation peaks are filled out. Consequently, the radiation intensity in this case mostly follows the pulse shape of the laser field producing a single gamma-radiation peak corresponding to the peak of the laser pulse. Note that the discussed qualitative features of the RAD for a sample electron do not depend on the initial position of the electron in the electron beam. The variation of the initial position introduces only a slight modification of a quantitative character. This will be discussed in the next section.

In addition to Fig. 3 of the paper, we show here in Fig. 2 the variations of the invariant laser field parameter ξ and the invariant parameter χ with respect to the laser phase $\bar{\eta} = \eta/(2\pi)$ in the case without SE. The laser and electron parameters are the same as in Fig. 3 of the paper. The peak of the laser fields is around $\bar{\eta} \approx 9$, as shown in Fig. 2(a). In addition, the peak of the radiation $\sim \chi$ is around $\bar{\eta} \approx 7$, as shown in Fig. 2(b). Besides, corresponding to Figs. 3(c) and 3(d) of the paper, the variations of the radiated photon energy ε_γ and the sample-electron energy ε_e with respect to the laser phase $\bar{\eta}$ are illustrated in Fig. 3. The first simulation of the radiation of the sample electron is shown in Figs. 3(a) and 3(c) corresponding to Fig. 3(c) of the paper, and the second simulation with the same laser and electron parameters is shown in Figs. 3(b) and 3(d) corresponding to Fig. 3(d) of the paper. Due to SE the two simulations represent much different results.

IV. THE RADIATION OF ELECTRONS WITH DIFFERENT INITIAL COORDINATES IN THE BEAM

According to Figs. 3(e)-3(h) in the paper, the radiation of a sample electron in other places of the electron beam is investigated. Figures 4 and 5 show the radiation of the sample electron located at $z = 0$ and $z = L_e$, respectively, of the electron-beam center with $x = y = 0$, and other parameters are the same as in Fig. 1. In both cases, with SE excluded, the radiation's angular distributions show the structure of the laser cycles, and the main gamma-photon peaks are located in the range of $11° \lesssim \theta \lesssim 45°$. Meanwhile, with SE included a single gamma-photon peak is formed in the radiation in the range of $11° \lesssim \theta \lesssim 45°$. As the electron moves from the front to the rear of the electron beam the radiation intensity decreases with the interacting laser fields because

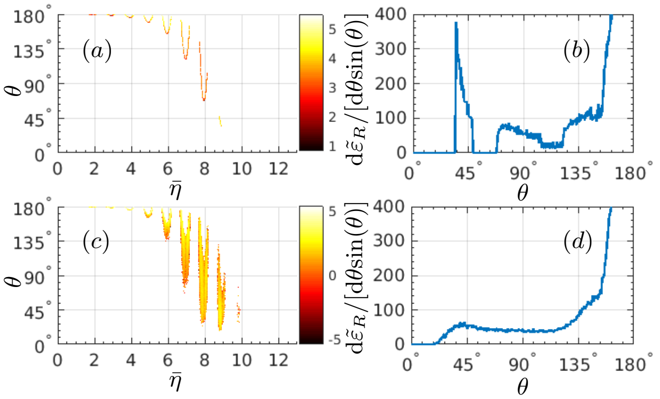

FIG. 6. The radiation's angular distribution excluding SE: (a) radiation intensity $\log_{10}[d^2\tilde{\varepsilon}_R/[d\bar{\eta}d\theta\sin(\theta)]]$ rad^{-1}, and (b) radiation energy $d\tilde{\varepsilon}_R/[d\theta\sin(\theta)]$ rad^{-1}, integrated over the azimuthal angle in the region of $-15° \leq \phi \leq +15°$. (c) and (d) are corresponding to (a) and (b), respectively, for the case including the SE. The initial coordinates of the sample electron are $z = L_e/2$ and $x = y = 0.5w_e$, and other electron and laser parameters are the same as in Fig. 1.

of the laser defocusing effects (since $\varepsilon_R \sim \chi \sim \xi\gamma$, as ξ decreases ε_R decreases as well).

The radiation of the sample electron beyond the electron-beam center with $z = L_e/2$ and $x = y = 0.5w_e$ is represented in Fig. 6. Since the electron is out of the electron-beam center, i.e., the propagation axis of the laser pulse, the laser fields experienced by the electron are much smaller than those at the electron-beam center (see Figs. 3(e)-3(h) in the paper), and consequently, the radiation angles of the gamma-photon peaks increase since $\theta_e = \theta_e^{(i)} - \delta\theta \sim 180° - \xi/\gamma$.

V. THE ROLE OF THE ELECTRON BEAM DENSITY

The SE signatures are also obtained with higher electron density. We show this explicitly by re-simulating the results of the paper with the use of a higher density. For instance, in Fig. 2(a) of the paper, the electron beam, with radius $w_e = \lambda_0$ and length $L_e = L_L = 6\lambda_0$, includes $2.4{\times}10^4$ electrons, and its density is $n_e \approx 10^{15}$cm^{-3}. Now, we re-simulate this case by employing $4.8{\times}10^4$ electrons (the density is 2 times higher). The comparison is illustrated in Fig. 7. As we know, the emitted photon number and intensity is proportional to the electron number, such that to facilitate the comparison the radiation of the high density case is divided by a factor of 2. In the high density case the same SE signatures are observed.

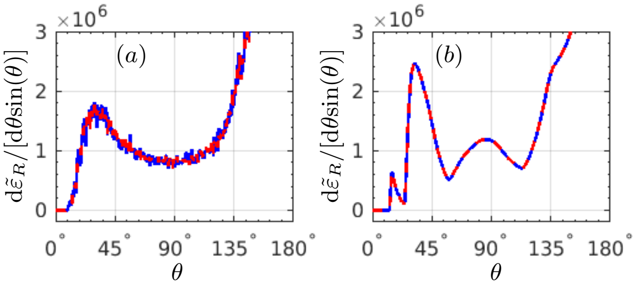

FIG. 7. The radiation's angular distribution $d\tilde{\varepsilon}_R/[d\theta\sin(\theta)]$: (a) including SE, and (b) excluding SE, respectively. The blue-solid curves present the results of employing 2.4×10^4 electrons, and the red-dashed curves present the ones of employing 4.8×10^4 electrons (higher density case). Other parameters are the same as in Fig. 1.

VI. COMPARISON BETWEEN THE "SOKOLOV" APPROACH AND THE "LANDAU-LIFSHITZ" APPROACH

We have carried out several simulations using Landau-Lifshits (LL) equation [49] in comparison with the Sokolov equation (SEQ). Generally, the LL equation is derived for the classical regime $\chi \ll 1$. Therefore, firstly we compare the LL and SEQ results in the classical regime of $\chi \ll 1$ and $\gamma \gg 1$. An example is shown in Fig. 8, where we compare the radiation's angular distributions simulated via the "Sokolov" approach (blue-solid curves) and with the "Landau-Lifshitz" approach (red-dashed curves) in the classical regime.

The "Sokolov" approach is the simulation method based on SEQ, which we employ in the manuscript as "no SE" method (excluding the stochasticity effects). In the "Landau-Lifshitz" approach in the applied ultrarelativistic regime, we keep only the leading term in γ in the LL radiation-reaction force, i.e., the third term in the LL radiation-reaction force in Eq. (1):

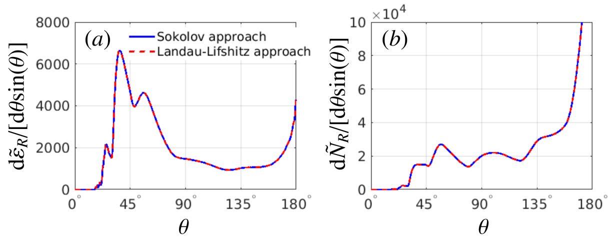

FIG. 8. The radiation's angular distribution in the classical regime: (a) radiation energy $d\tilde{\varepsilon}_R/[d\theta\sin(\theta)]$ (rad^{-1}), and (b) photon number $d\tilde{N}_R/[d\theta\sin(\theta)]$ (rad^{-1}), emitted in the region of $-15^\circ \leq \phi \leq +15^\circ$ of the azimuthal angle. $\varepsilon_0 = 10$ MeV, $\xi = 200$, and $\chi \lesssim 0.013$. Other parameters are the same as in Figs. 2(a) and 2(b) of the manuscript.

$$\begin{aligned}\boldsymbol{F}_{RR,classical} = \frac{2e^3}{3mc^3}\Bigg(\gamma\left(\left(\frac{\partial}{\partial t}+\frac{\boldsymbol{p}}{\gamma m}\cdot\nabla\right)\boldsymbol{E}+\frac{\boldsymbol{p}}{\gamma mc}\times\left(\frac{\partial}{\partial t}+\frac{\boldsymbol{p}}{\gamma m}\cdot\nabla\right)\boldsymbol{B}\right) \\ +\frac{e}{mc}\left(\boldsymbol{E}\times\boldsymbol{B}+\frac{1}{\gamma mc}\boldsymbol{B}\times(\boldsymbol{B}\times\boldsymbol{p})+\frac{1}{\gamma mc}\boldsymbol{E}\,(\boldsymbol{p}\cdot\boldsymbol{E})\right) \\ -\frac{e\gamma}{m^2c^2}\boldsymbol{p}\left(\left(\boldsymbol{E}+\frac{\boldsymbol{p}}{\gamma mc}\times\boldsymbol{B}\right)^2-\frac{1}{\gamma^2m^2c^2}\left(\boldsymbol{E}\cdot\boldsymbol{p}\right)^2\right)\Bigg),\end{aligned} \tag{1}$$

where γ, m, and e is the Lorentz factor, mass and charge of the electron, respectively, and c is the speed of the light in vacuum.

In quantum regime, as $\chi \lesssim 1$, the quantum recoil effects for the radiation intensity have to be included. The LL equation in its original form is not applicable in the quantum regime, however, heuristically one includes the recoil effects by renormalizing the radiation reaction force by the factor I_{QED}/I_C, the ratio of the radiation intensities within QED and classical approaches, which will account for the classical overestimation of the emitted radiation:

$$\boldsymbol{F}_{RR,quantum} = \frac{I_{QED}}{I_C}\boldsymbol{F}_{RR,classical}, \tag{2}$$

where,

$$I_{QED} = mc^2\int c\,(k\cdot k')\,\frac{dW_{fi}}{d\eta dr_0}dr_0, \tag{3}$$

$$I_C = \frac{2e^4E'^2}{3m^2c^3}, \tag{4}$$

W_{fi} is the radiation probability, $r_0 = \frac{(k\cdot k')}{\chi(k\cdot p_i)}$, and E' is the electric fields in the electron frame. k, k' and p_i is the four-vector of the wave vector of the driving laser, the wave vector of the radiation photon, and the momentum of the electron before the radiation, respectively.

In Fig. 9 an example of the radiation's angular distributions in the quantum regime is shown. All employed parameters in Fig. 9 are the same as in Figs. 2(a) and 2(b) of the manuscript.

The radiation's angular distributions demonstrate in Figs. 8 and 9 almost coincidence for SEQ and LL cases.

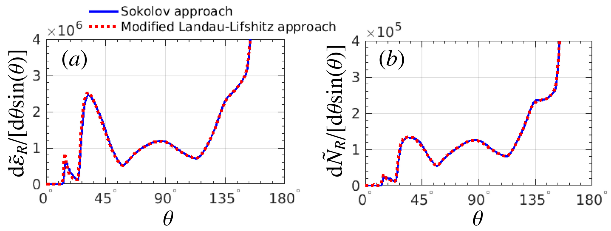

FIG. 9. The radiation's angular distribution in the quantum regime: (a) radiation energy $\mathrm{d}\tilde{\varepsilon}_R/[\mathrm{d}\theta\sin(\theta)]$ (rad^{-1}), and (b) photon number $\mathrm{d}\tilde{N}_R/[\mathrm{d}\theta\sin(\theta)]$ (rad^{-1}), emitted in the region of $-15^\circ \leq \phi \leq +15^\circ$ of the azimuthal angle. $\varepsilon_0 = 180$ MeV, $\xi = 600$ and $\chi \lesssim 1$. All parameters are the same as in Figs. 2(a) and 2(b) of the manuscript.

We can conclude that in the applied ultrarelativistic and weakly quantum regime the SEQ and the modified LL equation provide similar results. We prefer SEQ over the modified LL equation in the weakly quantum regime, because the first is derived systematically using energy-momentum conservation for the radiating electron in the ultrarelativistic weakly quantum regime, meanwhile the modified LL equation is a heuristic extrapolation of the LL equation out of its applicability region.
